# Vascular Response to Sildenafil Citrate in Aging and Age-Related Macular Degeneration

**DOI:** 10.1038/s41598-019-41509-2

**Published:** 2019-03-25

**Authors:** Glenn Yiu, Vivian S. Vuong, Steven Tran, Justin Migacz, David Cunefare, Sina Farsiu, Neha Khandelwal, Rupesh Agrawal, Chui Ming Gemmy Cheung

**Affiliations:** 1 Department of Ophthalmology & Vision Science, University of California, Davis, Sacramento, CA United States; 2 Rosalind Franklin University of Medicine and Science, North Chicago, IL United States; 3 Department of Biomedical Engineering, Duke University, Durham, NC United States; 4 National healthcare Group Eye Institute, Tan Tock Seng Hospital, Singapore, 308433 Singapore; 5 Singapore Eye Research Institute, Singapore National Eye Center 11 Third Hospital Avenue, Singapore, 168751 Singapore

## Abstract

Age-related macular degeneration (AMD) - the leading cause of vision loss in the elderly - share many risks factors as atherosclerosis, which exhibits loss of vascular compliance resulting from aging and oxidative stress. Here, we attempt to explore choroidal and retinal vascular compliance in patients with AMD by evaluating dynamic vascular changes using live ocular imaging following treatment with oral sildenafil citrate, a phosphodiesterase type 5 (PDE5) inhibitor and potent vasodilator. Enhanced-depth imaging optical coherence tomography (EDI-OCT) and OCT angiography (OCT-A) were performed on 46 eyes of 23 subjects, including 15 patients with non-exudative AMD in one eye and exudative AMD in the fellow eye, and 8 age-matched control subjects. Choroidal thickness, choroidal vascularity, and retinal vessel density were measured across the central macula at 1 and 3 hours after a 100 mg oral dose of sildenafil citrate. Baseline choroidal thickness was 172.1 ± 60.0 μm in non-exudative AMD eyes, 196.4 ± 89.8 μm in exudative AMD eyes, and 207.4 ± 77.7 μm in control eyes, with no difference between the 3 groups (P = 0.116). After sildenafil, choroidal thickness increased by 6.0% to 9.0% at 1 and 3 hours in all groups (P = 0.001–0.014). Eyes from older subjects were associated with choroidal thinning at baseline (P = 0.005) and showed less choroidal expansion at 1 hour and 3 hours after sildenafil (P = 0.001) regardless of AMD status (P = 0.666). The choroidal thickening appeared to be primarily attributed to expansion of the stroma rather than luminal component. Retinal vascular density remained unchanged after sildenafil in all 3 groups (P = 0.281–0.587). Together, our studies suggest that vascular response of the choroid to sildenafil decreases with age, but is not affected by the presence of non-exudative or exudative AMD, providing insight into changes in vessel compliance in aging and AMD.

## Introduction

The pathophysiology of age-related macular degeneration (AMD) is complex and multifactorial, owing to interactions between aging, genetic and environment factors. While proposed mechanisms of the disease have mainly included oxidative stress, immune dysregulation, and lipid/protein accumulation, a hemodynamic contribution to AMD has also been suggested^1,2^. Early work using laser doppler flowmetry demonstrated reduced choroidal blood flow in AMD eyes^3–5^. Histological studies also demonstrated loss of the choriocapillaris that may precede retinal pigment epithelial loss in choroidal neovascular membranes^6^. Although enhanced depth imaging optical coherence tomography (EDI-OCT) showed choroidal thinning with age but not early AMD^7–9^, choroidal thickness is reduced in eyes with reticular pseudodrusen and late-stage geographic atrophy^10–14^, or after anti-vascular endothelial growth factor (anti-VEGF) therapy^15–18^. To further understand the potential role of hemodynamics in AMD pathogenesis, we investigated if vascular compliance or stiffness may also be affected in eyes with this disease.

Systemic cardiovascular conditions such as atherosclerosis have similar risk factors as AMD, such as aging and smoking, and are associated with loss of vascular compliance^19^. Increased oxidative stress has been associated with increased arterial stiffness^20^, while age-related changes in elastin and collagen in vessel walls contribute to loss of vascular compliance^19^. Decreased arterial compliance is correlated with a higher risk of cardiovascular disease, and has been implicated in erectile dysfunction as well^21,22^, although an association with AMD has never been demonstrated. Interestingly, erectile dysfunction is commonly treated with sildenafil citrate (Viagra; Pfizer, New York), a selective inhibitor of cyclic guanosine monophosphate (cGMP)-specific phosphodiesterase type 5 (PDE5) which reduces arterial stiffness and promotes vasodilation^21^. Sildenafil has also been shown to increase choroidal thickness^23,24^ and promote retinal vasodilation in in young, healthy subjects^25^.

In this prospective study, we employed multimodal OCT imaging including EDI-OCT and OCT-angiography (OCT-A) to investigate choroidal and retinal vascular response to a single-dose of oral sildenafil citrate in eyes with AMD and those of age-matched control subjects. Vascular compliance is directly related to vessel area, and inversely related to pressure. Since sildenafil citrate at its maximum therapeutic dose of 100 mg does not produce any significant change in intraocular pressure or mean systemic blood pressure^26,27^ - the two determinants of ocular perfusion pressure - we hypothesize that the change in vessel size induced by sildenafil may serve as an indicator of vascular compliance or stiffness. Here, we selected patients with unilateral exudative AMD and non-exudative AMD in the fellow eye, as well as age-matched controls, to evaluate potential differences in vessel compliance. Using cross-sectional EDI-OCT to measure choroidal thickness and vascularity, and en-face OCT-A to assess retinal vascular density, we examined whether vascular response to sildenafil may be compromised in eyes with AMD, and whether such an impact may be an indicator of reduced vascular compliance.

## Methods

### Subject selection and study design

This study was approved by the Institutional Review Board of University of California, Davis and was conducted in accordance with the tenets of the Declaration of Helsinki. Informed consent was obtained from all patients who took part in this study. Subjects were selected if they were 65 years or older and have been diagnosed with non-exudative AMD in one eye and exudative AMD in the fellow eye, or are age-matched control subjects with no known ocular disease. Study eyes with history of any other ocular pathologies or ocular surgeries, with the exception of uncomplicated cataract extraction surgery, were excluded from enrollment. Eyes with high myopia greater than 6 diopters (D) spherical equivalent were excluded, due to the association of high myopia with choroidal thinning. Subjects who currently use any oral PDE5 inhibitors or systemic corticosteroids, have a history of uncontrolled diabetes or hypertension, or have any contraindication to sildenafil use including history of cardiovascular disease or stroke, hepatic cirrhosis, severe renal impairment, anatomical deformation of the penis, disorders predisposing to priapism, or current use of organic nitrates, alpha-blockers, or potent cytochrome P450 3A4 inhibitors were all excluded. Clinical charts were reviewed to collect demographic and clinical data, including age, sex, baseline best-corrected visual acuity (logMAR), refractive error (D), intraocular pressure (mmHg), and lens status (phakic or pseudophakic), as well as AMD status (exudative or non-exudative), including any history of anti-VEGF therapy or the presence of geographic atrophy (GA). Enrolled subjects were given a single oral dose of 100 mg sildenafil citrate, and EDI-OCT and OCT-A imaging were performed before, and at 1 and 3 hours after treatment, based on published time-course studies of choroidal thickening using sildenafil in young, healthy subjects.

### EDI-OCT imaging

EDI-OCT images were obtained using the 40 kHz Spectralis spectral-domain OCT (SD-OCT) unit (Heidelberg Engineering, Heidelberg, Germany). A single 30-degree horizontal line scan (approximately 9 mm) consisting of 768 A-scans per B scan and centered on the fovea was taken in high-speed EDI-mode. EDI-OCT images before and after sildenafil administration were performed in the same manner, with retinal tracking to ensure the same scan location at each time point.

### Choroidal thickness measurement

**Figure 1:**
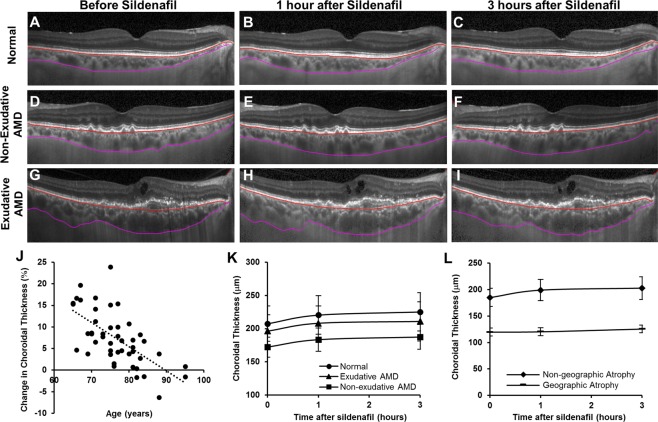
Representative enhanced-depth imaging OCT images of the central macula showing the anterior (red) and posterior (magenta) boundaries of the choroid segmentation in eyes from a normal age-matched patient (**A**–**C**), with non-exudative AMD (**D**–**F**), and with exudative AMD (**G**–**I**), before (**A**,**D**,**G**) and at 1 hour (**B**,**E**,**H**) and 3 hours (**C**,**F**,**I**) after a 100 mg oral dose of sildenafil citrate. A scatterplot demonstrates an inverse association between age and the amount of choroidal thickness change (%) at 3 hours after sildenafil treatment (**J**). Line graphs show an increase in choroidal thickness after sildenafil in both normal and AMD eyes (**K**), but no detectable choroidal thickening in eyes with geographic atrophy (**L**).

Choroidal thickness in the central macula was measured from EDI-OCT images using the Duke Optical Coherence Tomography Retinal Analysis Program (DOCTRAP, version 62.0), a custom semi-automatic image segmentation software designed using MATLAB (Mathworks)^28^. The inner boundary was automatically determined as the outer border of the hyperreflective line corresponding to the retinal pigment epithelium (RPE)-Bruchs membrane complex with manual adjustments where needed^29^, while the outer boundary was manually adjusted by a trained masked grader to the outer border of the choroid stroma, which has been shown previously to demonstrate the highest reliability for choroidal thickness measurements^30–32^. Choroidal thickness was measured by averaging measurements from every A-scan position across the central 3 mm segment around the fovea along the horizontal line scan, which provides a more reproducible choroidal thickness measurement than a single location (Fig. 1)^31^.

### Choroidal vascularity measurement

**Figure 2:**
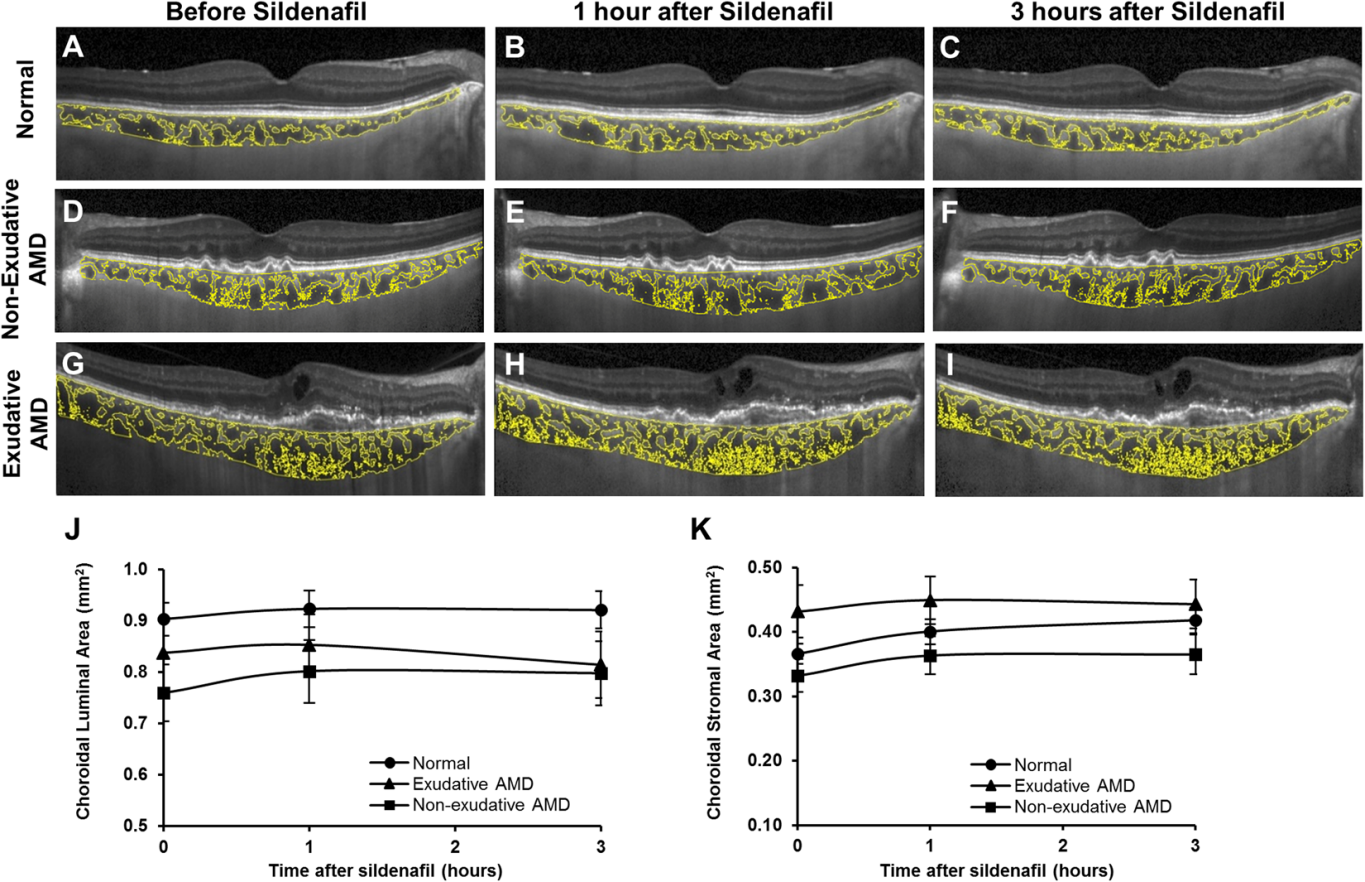
Representative enhanced-depth imaging OCT images of the central macula showing post-binarization overlay of the region of interest on the scans demonstrating the luminal and stromal areas (yellow) in eyes from a normal age-matched patient (**A**–**C**), with non-exudative AMD (**D**–**F**) and with exudative AMD (**G**–**I**), before (**A**,**D**,**G**), and at 1 hour (**B**,**E**,**H**) and 3 hours (**C**,**F**,**I**) after a 100 mg oral dose of sildenafil citrate. Line graphs show a greater relative increase in stromal area (**K**) than luminal area (**J**) in the choroid.

To determine the relative contributions of vascular and stromal components to choroidal thickness measurements, we measured the choroidal luminal area (LA) and stromal area (SA) using a protocol validated in published studies^33–35^. Images were uploaded onto a publicly-available software - Fiji (ImageJ, National Institutes of Health, Bethesda, MD), and the polygon tool was used to select the region of interest (ROI), using criteria similar to those for determining choroidal thickness to select the inner and outer boundaries of the choroid. Images were converted to 8-bit to allow auto-thresholding using the Niblack method^36^ for better resolution and demarcation of the LA and SA. The image adjusted by automatic local thresholding was then converted to a red, green, blue (RGB) image and the LA was determined using the color threshold tool (Fig. 2). We also measured the choroidal vascularity index (CVI), which was computed as LA/(LA + SA).

### OCT-angiography imaging

For evaluating retinal vessels, OCT-A was performed using the 68 kHz Cirrus HD-5000 OCT device (Carl Zeiss Meditec). A 6 × 6 mm cube scan consisting of 350 B-scans and 350 A-scans per B-scan, centered on the fovea, was acquired along with en-face structural images and three-dimensional microvascular maps based on the optical microangiography (OMAG) complex algorithm that uses amplitude and phase aspects of the OCT signal to extract *in vivo* blood flow information. OCT-A images before and after sildenafil administration were performed in the same manner, with retinal tracking to ensure the same scan location at each time point.

### Retinal vascular density measurement

**Figure 3:**
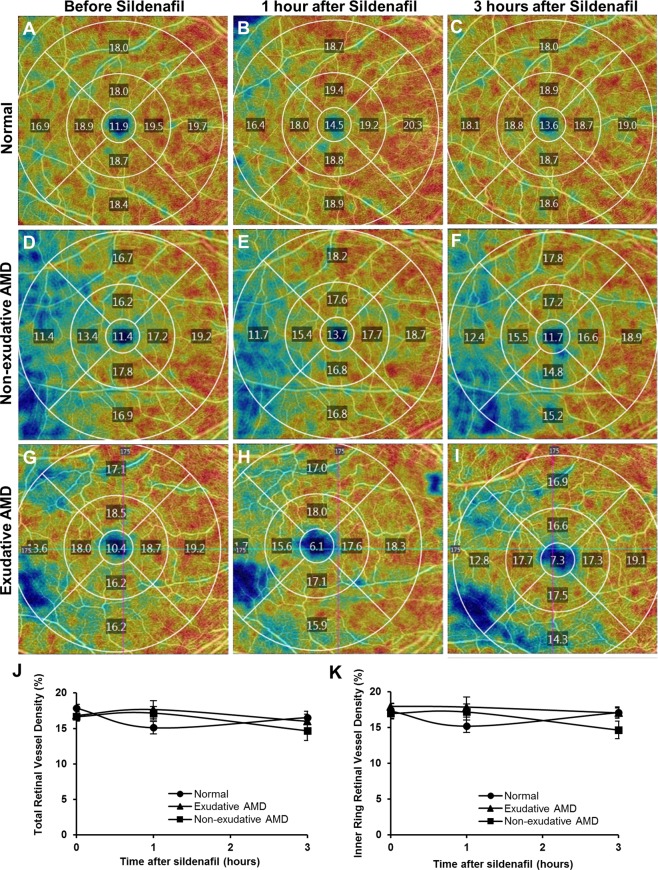
Representative en-face OCT-A images of the central macula showing superficial retinal vessel density values measured from the central 1 mm diameter circle, and each quadrant of the 1–3 mm diameter inner ring and 3–6 mm diameter outer ring of the Early Treatment of Diabetic Retinopathy Study grid, in eyes taken a normal age-matched patient (**A**–**C**), with non-exudative AMD (**D**–**F**) and with exudative AMD (**G**–**I**), before (**A**,**D**,**G**), and at 1 hour (**B**,**E**,**H**) and 3 hours (**C**,**F**,**I**) after a 100 mg oral dose of sildenafil citrate. Line graphs show no significant change in the total retinal vessel density (**J**) or inner ring retinal vessel density (**K**) after sildenafil in either normal or AMD eyes.

Retinal vascular density was measured from the OCT-A images using AngioPlex investigational software (version 10.0.0.12787), which measures the ratio of the total vessel area to the total area of interest, captured from the superficial retinal slab automatically segmented from the internal limiting membrane to the inner plexiform layer, within a 6 mm-diameter circle centered on the fovea, and from the 3 mm-diameter inner ring (Fig. 3). Because the OCT-A signal only measures vessels where there is blood flow, the retinal vascular density measurement excludes nonperfused vessels.

### Statistical analyses

The association between AMD status and age with baseline choroidal thickness, choroidal vascularity (stromal area, luminal area, and CVI), and retinal vascular density were evaluated using linear regression analyses with generalized estimating equations to account for up to two eyes per subject. The association of GA with these parameters in non-exudative AMD eyes was assessed by independent samples t-tests, while relationship with number of anti-VEGF treatments in eyes with exudative AMD was determined by linear regression analyses. Change in vascular measures over time after sildenafil administration was assessed with a repeated-measures ANOVA with pairwise comparisons to separately determine within-subject (time) and between-subject (AMD type) effects. P-values less than 0.05 were considered to be statistically significant. All statistical analyses were performed using SPSS software (version 25, IBM).

## Results

### Demographics and clinical characteristics

A total of 46 eyes from 23 subjects were included in the analysis. Among the participants, 15 subjects had non-exudative AMD in one eye and exudative AMD in the fellow eye, and 8 age-matched control subjects had no known retinal or choroidal diseases. Three of the eyes with non-exudative AMD also had signs of geographic atrophy. Most of the eyes with exudative AMD had received prior intravitreal anti-VEGF therapy, including ranibizumab, bevacizumab, and/or aflibercept, with a median of 9 treatments (range 3–55). The mean age for AMD subjects was 78.0 ± 8.1 years, with 3 men and 12 women. For age-matched control subjects, mean age was 73.6 ± 4.6 years, with 3 man and 5 women. Mean best-corrected visual acuity was 0.270 ± 0.225 logMAR (Snellen equivalent 20/37) for non-exudative AMD eyes, 0.367 ± 0.227 logMAR (Snellen equivalent 20/47) for exudative AMD eyes, and 0.187 ± 0.178 logMAR (Snellen equivalent 20/31) for control eyes. There was no significant difference in mean refractive errors between eyes with non-exudative AMD, exudative AMD, and healthy controls (P = 0.323).

### Choroidal thickness after sildenafil

Older age of subjects was associated with thinner choroid at baseline (P = 0.005), consistent with prior reports^37^. Mean choroidal thickness at baseline was 172.1 ± 60.0 μm in non-exudative AMD eyes, 196.4 ± 89.8 μm in exudative AMD eyes, and 207.4 ± 77.7 μm in control eyes, with no difference between the 3 groups (P = 0.116) when adjusted for age.

After sildenafil administration, choroidal thickness of normal eyes increased by 6.3% at 1 hour (13.0 μm, P < 0.001) and 8.6% (17.8 μm, P < 0.001) at 3 hours (Fig. 1A–C). Eyes with non-exudative AMD showed choroidal thickening of 6.6% (11.4 μm, P = 0.002) at 1 hour, and 9.0% (15.4 μm, P = 0.002) at 3 hours (Fig. 1D–F), while eyes with exudative AMD showed choroidal thickening of 6.0% (11.8 μm, P = 0.001) and 7.4% (14.6 μm, P = 0.014) at 1 and 3 hours after sildenafil (Fig. 1G–I, Table 1). Eyes from older subjects showed less choroidal expansion (P = 0.001, Fig. 1J), but there was no significant difference between normal eyes or eyes with either form of AMD (P = 0.464), even after adjustment for age (P = 0.666; Fig. 1K).

**Table 1:** Choroidal and retinal vascular changes after sildenafil citrate in AMD and normal eyes.

	Age-matched normal eyes (n = 16)	Non-exudative AMD eyes (n = 15)	Exudative AMD eyes (n = 15)	P-value*
**Choroidal thickness (mm)**				0.464
Baseline	207.5 ± 77.7	172.1 ± 60.0	196.4 ± 89.8	
1 hour	220.5 ± 82.5	183.5 ± 69.3	208.2 ± 95.4	
3 hour	225.3 ± 81.7	187.6 ± 73.6	211.0 ± 108.0	
**Choroidal luminal area (mm** ^2^ **)**				0.203
Baseline	0.90 ± 0.12	0.76 ± 0.19	0.84 ± 0.24	
1 hour	0.92 ± 0.14	0.80 ± 0.21	0.85 ± 0.21	
3 hour	0.92 ± 0.14	0.80 ± 0.22	0.81 ± 0.22	
**Choroidal stromal area (mm** ^2^ **)**				0.109
Baseline	0.37 ± 0.06	0.33 ± 0.09	0.43 ± 0.14	
1 hour	0.40 ± 0.07	0.36 ± 0.10	0.45 ± 0.13	
3 hour	0.42 ± 0.07	0.37 ± 0.11	0.44 ± 0.13	
**Total retinal vascular density (%)**				0.281
Baseline	17.7 ± 1.8	13.9 ± 6.9	16.3 ± 1.9	
1 hour	15.1 ± 3.0	14.3 ± 7.3	16.7 ± 3.0	
3 hour	16.7 ± 2.9	12.4 ± 6.0	15.6 ± 1.9	
**Inner circle retinal vascular density (%)**				0.587
Baseline	17.2 ± 3.5	17.0 ± 1.6	17.0 ± 2.2	
1 hour	15.2 ± 3.0	17.2 ± 2.5	17.0 ± 3.1	
3 hour	17.1 ± 2.6	14.6 ± 2.5	16.5 ± 1.8	

^*^P-value based on repeated measures ANOVA with time as within-subject and AMD type as between-subject variables.

In eyes with non-exudative AMD, the presence of GA was associated with choroidal thinning at baseline compared with eyes without GA (119.9 ± 13.3 μm vs. 185.2 ± 60.2 μm, P = 0.004), and showed a trend toward slower choroidal expansion after sildenafil treatment (0.6 ± 1.2% vs. 6.8 ± 4.9% at 1 hour and 5.0 ± 1.6% vs. 8.1 ± 7.1% at 3 hours, P = 0.090) (Fig. 1L), although this association was not statistically significant when adjusted for age (P = 0.309). This may be attributed to the fact that the subjects with GA were older (mean age 84.3 ± 3.5 years vs. 76.4 ± 8.2 years), and older eyes undergo less choroidal expansion after sildenafil treatment (Fig. 1J). In eyes with exudative AMD, the number of previous anti-VEGF injections was not associated with choroidal thickness at baseline (R^2^ = −0.077, P = 0.961), and did not impact choroidal thickening after sildenafil (P = 0.707), even when adjusted for age (P = 0.520). Together, these results suggest that both choroidal thickness and choroidal response to sildenafil decreases with age, but does not appear to be impacted by the presence of exudative or non-exudative AMD. Eyes with GA may exhibit less change choroidal thickness likely due to the older age of this subgroup and the thinner choroid associated with this disease phenotype.

### Choroidal vascularity after sildenafil

Because the choroid consists of both vascular and stromal components, we sought to evaluate if the choroidal expansion after sildenafil treatment was attributed to changes in vessel lumen or extravascular stroma (Fig. 2A–I). At baseline, older age was associated with reduced luminal (P = 0.003) and stromal area (P = 0.004), but not CVI (P = 0.509). Eyes with exudative AMD had greater stromal area and lower CVI than healthy eyes or eyes with nonexudative AMD (P = 0.001 and P < 0.001, respectively), consistent with published reports demonstrating reduced choroidal vascularity of eyes with exudative AMD^38^. We then measured the luminal and stromal areas of the choroid after sildenafil treatment and found that the choroidal thickening was primarily attributed to the expansion of the stromal component rather than vessel lumina (Fig. 2J,K), but neither measure was significantly impacted by AMD status (P = 0.203 & 0.109, Table 1). These findings suggest that the impact of sildenafil on the vascular or stromal components of the choroid are reduced with age, but not the presence of AMD.

### Retinal vascular density after sildenafil

To explore the impact of sildenafil on retinal vessels, we measured the superficial retinal vascular density from the central macula across a 6 mm circle (Fig. 3A–J), and found no significant difference in total retinal vessel density at baseline between normal eyes and eyes with either form of AMD (P = 0.104), and no significant change in vessel density between the 3 groups after sildenafil treatment (P = 0.281). We also measured retinal vascular density from the 3 mm inner circle to exclude the foveal avascular zone and outer ring, which may be affected by decentration artifacts (e.g. Fig. 3H), and again found no significant difference in baseline retinal vessel density (P = 0.983) or vessel density change after sildenafil (P = 0.587). Together, these results suggest that sildenafil treatment does not affect retinal vascular density, or at least not to a detectable degree using this OCT-A system.

## Discussion

In this study, we employed a combination of cross-sectional EDI-OCT and en-face OCT-A images to evaluate choroidal and retinal vascular changes after a single administration of an oral phosphodiesterase inhibitor to better understand the possible role of vascular compliance in AMD and age-matched control eyes. We found that the choroidal thickening in response to sildenafil is reduced with older age, but does not appear to be affected the presence of either non-exudative or exudative AMD. We also found that the stromal components of the choroid accounts for most of the choroidal expansion after sildenafil treatment, but also does not appear to be impacted by AMD status. Finally, we did not detect any change in retinal vascular density in the central macula, and thus could not draw any conclusion on retinal vessel compliance.

Previous studies have evaluated the effect of sildenafil on the choroid in young, healthy subjects using EDI-OCT. Kim *et al*. showed a 9.3% to 11.6% increase in choroidal thickness 2 hours after a 50 mg dose^23^. Vance and colleagues also showed a 12.3% choroidal thickening at 1 hour after 100 mg of sildenafil^24^. Interestingly, our study using older patients demonstrated only a 6.0% to 8.6% increase in choroidal thickness 1 to 3 hours after a 100 mg dose of the drug, even in eyes without AMD, suggesting that aging alone may lead to greater vascular stiffness in the choroid. Consistent with this notion, we found in our cohort that the degree of choroidal thickening after sildenafil was inversely-associated with age (Fig. 1J), suggesting that aging is an important determinant of choroidal vascular compliance.

Although we detected no significant difference in choroidal response between normal and AMD subjects, we did find a trend toward slower choroidal expansion in eyes with GA. This difference was not significant after adjusting for age, likely due to the small number of patients in our cohort with GA, so it is difficult to conclude if the presence of GA independently reduces choroidal vascular compliance, or if the effect is primarily due to the older age and thinner choroid in these patients. These findings are similar to our prior study of eyes from the Age-Related Eye Disease Study 2 (AREDS2) which showed no correlation between choroidal thickness and AMD status after adjusting for age, but subgroup analysis showed choroidal thinning in the GA cohort^7^. Whether changes in choroidal thickness or compliance are causative or a result of GA pathophysiology remains unknown, and will be an area of interest for future inquiry.

In addition to measuring choroidal thickness, we also assessed choroidal vascularity in our study, which has been shown to exhibit less variability and less influence by physiologic factors^35^. We found that baseline CVI was significantly lower in eyes with exudative AMD when compared with normal or nonexudative AMD eyes, as previously described^38^, but sildenafil treatment did not impact choroidal vascularity differently based on AMD status, suggesting that AMD may not affect the compliance of either the stromal or vascular components of the choroid. Interestingly, we found that the stromal component of the choroid accounts for most of the choroidal thickening after sildenafil treatment, possibly highlighting the role of extravascular smooth muscle cells in regulating dynamic changes in choroidal thickness and blood flow. Our finding may also explain some published reports showing no significant change in choroidal blood flow after sildenafil treatment^26,39^.

In contrast to our observations in the choroidal circulation, our study also showed no significant change in retinal vascular density in response to oral sildenafil. These results are consistent with prior studies which showed no clear dilation of retinal vessel caliber after sildenafil using monochromatic fundus photography^40^, although some reports suggest that the major retinal veins may become dilated in both normal and AMD subjects^41^. The difference between the retinal and choroidal vascular response to sildenafil we observed may be attributed to differences in autoregulation - choroidal vessels have higher blood flow but lack autoregulation, while retinal vessels lacks autonomic innervation but shows efficient autoregulation^42^. However, some evidence suggests that the choroid may also exhibit autoregulatory behavior^43,44^ and potentially dampen the choroidal response after oral sildenafil, although role of nitric oxide pathways in choroidal autoregulation remains to be explored. Finally, the resolution of our OCT-A system may be too low to detect changes in smaller-diameter retinal vessels compared to the use of cross-sectional OCT B-scans to measure larger choroidal vessels. Using more advanced OCT-A technologies in the future may allow more refined analyses of vascular compliance in the eye.

Although the hemodynamic contribution to AMD pathogenesis remains unclear, some researchers have evaluated the potential use of PDE inhibitors as a potential strategy for therapy. Intravenous delivery of moxaverine, a non-selective PDE inhibitor, demonstrated increased choroidal blood flow using laser-doppler flowmetry in both AMD and control subject eyes^45^. However, while studies using oral sildenafil have been shown to increase choroidal thickness^23^, changes in choroidal blood flow has not been reliably demonstrated in either normal or AMD subjects^26,39^. In addition, while a hemodynamic role in the pathogenesis of AMD has been postulated^1^, it is unclear if increasing choroidal blood flow would actually impact the development or progression of AMD^3–5^. Given the lack of current evidence for benefit and known cardiovascular and ocular risks of sildenafil such as non-arteritic ischemic optic neuropathy^46^, the use of PDE inhibitors as a therapy for AMD should await further evaluation.

The strengths of our study include the use of live, multimodal ocular imaging in a prospective study to measure dynamic vascular changes in response to a PDE5 inhibitor as a surrogate measure of vascular compliance. In contrast to retrospective, cross-sectional analyses that assessed vascular abnormalities in eyes with AMD, our analysis provide insight into vessel stiffness and potential effects on vascular flow. The use of AMD subjects with exudative AMD in one eye and non-exudative AMD in the fellow eye excludes the influence of genetic and environmental factors in comparing the two subtypes of AMD. However, our results are also limited by the small sample size, and the variable prior use of anti-VEGF in the eyes with exudative AMD, which has been shown to cause choroidal thinning with repeated treatments^15,47^. Also, the vascular response to sildenafil is primarily pharmacologic rather than mechanical, and we assumed that ocular perfusion pressure does not change significantly over the time course of our study based on previous reports^26,27^. Nevertheless, our results suggest that choroidal vascular compliance may decrease with age, and that eyes with AMD may not show any significant difference in choroidal vascular stiffness. These studies may help us gain additional insight into a potential hemodynamic role to the pathogenesis of AMD and aging.
